# Plaster Impressions

**Published:** 1860-06

**Authors:** S. Clippenger


### PLASTER IMPRESSIONS.

BY DR. S. CLIPPENGER.

Read at the April meeting of the Mad River Valley Dental Association.

THIS may be a very commonplace subject, yet a very important one—second to none, at least, in mechanical dentistry. A correct impression of the mouth is as important to a successful dental structure, as a healthy and well organized brain is to a good *mind*, or a firm foundation to the lofty tower. Without these pre-requisites they will all fail alike to accomplish the end sought for in the outset. Then, let our *motto* be—start right and make every effort to keep right; for without the former we will be as the mariner without his compass, or the unregenerated man who is without the beacon star of heaven to guide him through the journey of time, to the realms of bliss, for they will all fail to accomplish the end for which they were designed. It is important, then, that our material be of the right kind, and of the *best* quality; for both are indispensable to success.

The first thing is to have properly calcined plaster of Paris that has gone through No. 10 bolting cloth, with cup, spatulæ and water and impression cups at hand. Our patient in the chair, (suppose a lady) we instruct her to sit erect, with chin down, and the head inclined forward, to prevent the plaster and saliva from flowing down the throat, thereby preventing the unpleasantness of gagging the patient and spoiling the impression. A napkin should be previously placed over the chest. If the saliva is of a ropy nature, we have the patient rinse her mouth with cold water. We are now ready to mix our plaster and take the impression. We have the plaster of the consistence of thick cream, and stir it till it is free from bubbles, then fill our impression cup and take our position behind and above the patient, so that our waist is as high as her head. Stooping over her, we place the cup in her mouth, holding it with the thumb and forefinger of the right hand, using the index finger of the *left* hand to lift and relieve the lip and cheeks from the cup, using the nose for our guide-board to tell when it is straight. We bring the cup up first in front, gradually bringing the back portion up, thereby preventing air-cells in the impression of a deep palate. After the cup is brought up to its place, we raise the lip and cheeks, in order to let the plaster flow up over the gum. Then placing the index fingers one on each side of the cup, we hold it firm till the plaster sets, which will require from three to five minutes. We then tell our patient to close her lips, (being careful not to let her press the cup with the lower jaw) and fill her mouth with air, which will be forced under the impression, so that we can remove it with ease, unless there are prominences and depressions on the outside of the alveolar ridge, when it will have to be aided by pressing the front portion of the cup upward, which will force the back portion down. Then remove the cup, and if any of the impression is broken off, we are careful to examine the mouth and get the pieces and set them to their places on the impression. If necessary we fasten them with plaster that they may retain their places while filling for a positive cast. It is thought, by those not accustomed to taking impressions with plaster, that an impression of the lower jaw is difficult, if not *impossible* to be taken. This is a mistake. It is true that it requires more care than the upper, but is not any more unsuccessful. We mix our plaster, fill the cup as for the upper, and, taking our position as before, turn the cup over quickly, holding it in the right hand, with thumb and finger, using the index finger of the left hand to relieve the lip and cheeks from the cup, and place the cup in, using the nose as our guide. We are careful to see that the lip and cheeks are kept out, so as not to get under the plaster; and have the patient to keep the tongue back as much as possible. Now we press the cup down first in the back portion, gradually bringing it down in front, so as to keep the plaster in the front portion of the mouth. Then we hold it firm to its place, by putting our thumbs, one on each side, letting the fingers pass under the jaw, we keep it to its place till the plaster sets. Then remove it as before, only press downward instead of upward, so as to start it in the back part first.

If we observe the above precautions, we can not fail to get a true cast; but if we fail to take these, we may expect to fail in getting a correct impression. It *always* requires care on the part of both patient and operator: but if that is done we have the satisfaction of knowing that we have a correct start for our dental structure, one that can not be pressed out of shape without our knowing it, as may be the case with other materials used for that purpose; for if it is broken we set it up again, and go on as if nothing had happened, feeling assured that we have a cast that is a fac simile of that portion of the mouth designed for our purpose. If the gums, palate, or any portion of the mouth from which we wish an impression, be soft or spongy, it does not press that portion out of shape, but we have a correct cast, which we all know is indispensable to success in fitting the mouth with a plate, let that be of whatever material or composition you may choose. Well, says one, *I* can take just as good an impression with *wax* as *you* can with plaster. That we think is impossible. My friend, have you ever tried plaster? Yes, two or three times only. Well, that is just often enough to set you against the use of it; for you would be excited all the time, minding every thing that you ought not to, and thinking it would probably be a failure. You were destitute of *faith;* and all you could do would just make a muss, and a failure was the consequence. You must remember that without faith there are no works that are successful.

I have tried both, and find plaster to excel wax, as far as gold excels amalgam in preserving a tooth. With it your impression is perfect. It settles to every part without changing them in the least. As far as its being *filthy*, there is not a particle of filth about it; nor is there any sickening taste to it, for it has no taste at all; and, with proper care and experience, you will be able to avoid all the muss that you so much dread, unless you are one of those would-be operators that make a muss out of every thing they undertake to do. Then, you are a hopeless case, unless we can get you to do your first work over, and learn anew. Then, if you have a proper *tutor*, you may learn to keep your hands and person clean, as well as your instruments, napkins, and other appliances, such as spittoon, glass pitcher, and your *mouth* which is *last* but not *least* important by any means.

